# Progressive Multifocal Leukoencephalopathy in HIV-Infected Children: A Case Report and Literature Review

**DOI:** 10.1155/2009/348507

**Published:** 2009-09-16

**Authors:** Peninnah Oberdorfer, Charles H. Washington, Kamornwan Katanyuwong, Podjanee Jittamala

**Affiliations:** ^1^Division of Infectious Diseases, Department of Pediatrics, Faculty of Medicine, Chiang Mai University, Chiang Mai 50200, Thailand; ^2^Department of Epidemiology, Bloomberg School of Public Health, Johns Hopkins University, Baltimore, MD 21205, USA; ^3^Division of Neurology, Department of Pediatrics, Faculty of Medicine, Chiang Mai University, Chiang Mai 50200, Thailand

## Abstract

We report a case of a perinatally HIV-infected patient aged 9 years, who presented with right-sided hemiplegia. His initial CD4 T-cell was of 0.21% (4 cells/*μ*L) and plasma HIV RNA virus of 185 976 copies/mL (log 5.27). Plasma and CSF samples were subsequently positive for JCV. Twelve days after the initiation of highly active antiretroviral therapy (HAART), the MRI showed progressive white matter lesions with asymmetrical deep and subcortical white matter lesions over the left frontotemporoparietal region and the right frontal lobe. Immune Reconstitution Inflammatory Syndrome (IRIS) was suspected, and the patient was treated with methylprednisolone. His clinical symptoms worsened and despite therapy the patient deteriorated.

## 1. Background

Progressive multifocal leukoencephalopathy (PML) is caused by the JC virus (JCV). As a demyelinating disease, PML typically presents with altered mental status, motor deficits, and ataxia, and is associated with immunosuppression, especially human immunodeficiency virus (HIV) [1]. Prevalence of JCV specific antibodies increases rapidly during childhood, but the mode of transmission is unknown [2]. PML is rare in HIV-infected children and even more uncommonly associated to immune reconstitution inflammatory syndrome (IRIS) in children.

## 2. Case Report

### 2.1. First Admission

A previously healthy 9-year-old boy (whose mother was recently found to be HIV-positive) presented to the hospital with 1 week of right-sided hemiplegia and right-sided facial palsy. Past medical history included psoriasis, diagnosed 4 years prior. His only HIV exposure was perinatal. On exam the patient weighed 22 kilograms, and vitals signs were within normal limits. He was alert and oriented with a normal level of consciousness and responded appropriately to questions. His speech was slowed and slurred, but this was his baseline according to his mother. He had right-sided facial palsy and right-sided tongue deviation; otherwise cranial nerves were intact. The boy had 3/5 strength in the right upper extremity and 4/5 strength in the right lower extremity. Left-sided strength was 5/5. The patient was able to walk with limited difficulty. Deep tendon reflexes were 2+ and 3+ throughout. Babinski's sign showed dorsiflexion of the right 1st toe. Sensation was intact throughout. The patient also had clusters of 1-2 mm skin colored papules on his forehead and left cheek in addition to diffuse mild psoriatic scaling. Immunizations were up to date. The patient was admitted for a workup of these symptoms.

Laboratory studies showed complete blood count: hematocrit 31.6%, hemoglobin 10.5 g/dL, white blood cell count 5000 cells/*μ*L (N 41%, L 35%, E 12%, M 6%, B 1%, atypical L 1%), and 157 000 platelets/*μ*L. HIV-antibody test was positive with a CD4 T-cell 0.21% (4 cells/*μ*L) and plasma HIV RNA virus of 185 976 copies/mL (log 5.27). Tests for Cryptococcus antigen, Toxoplasmosis antigen, Ebstein Barr virus IgG and IgM, Cytomegalovirus IgG and IgM, Hepes Simplex virus polymerase chain reaction (PCR), Japanese encephalitis as well as cultures for tuberculosis and fungi (plasma and cerebrospinal fluid, CSF) were all negative. Plasma and CSF samples were positive for JCV by real time PCR with a plasma RNA level of 226 copies/mL.

**Figure 1:**
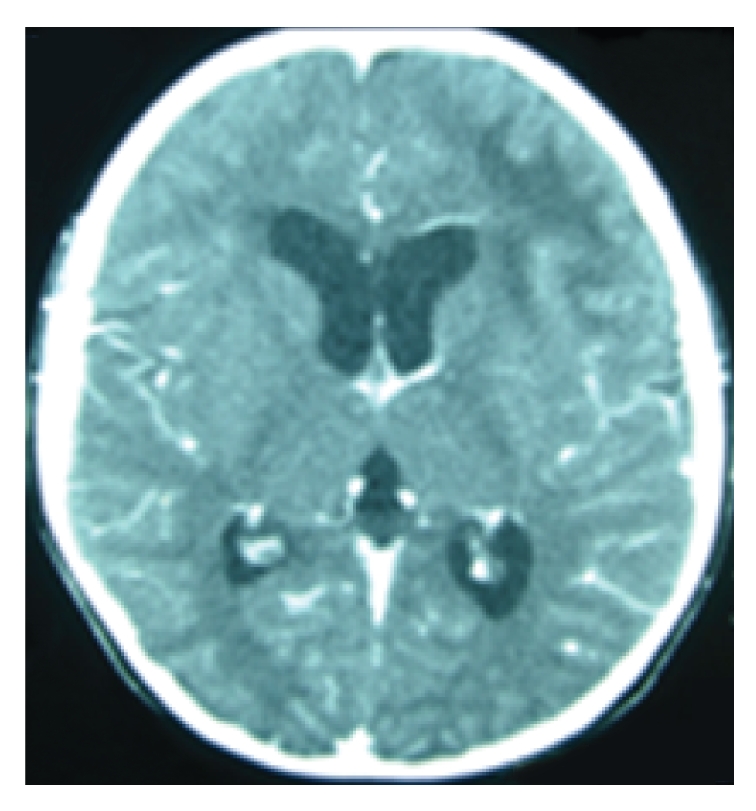
CT on day 30 of onset of symptoms and 1 week post-HAART initiation.

**Figure 2:**
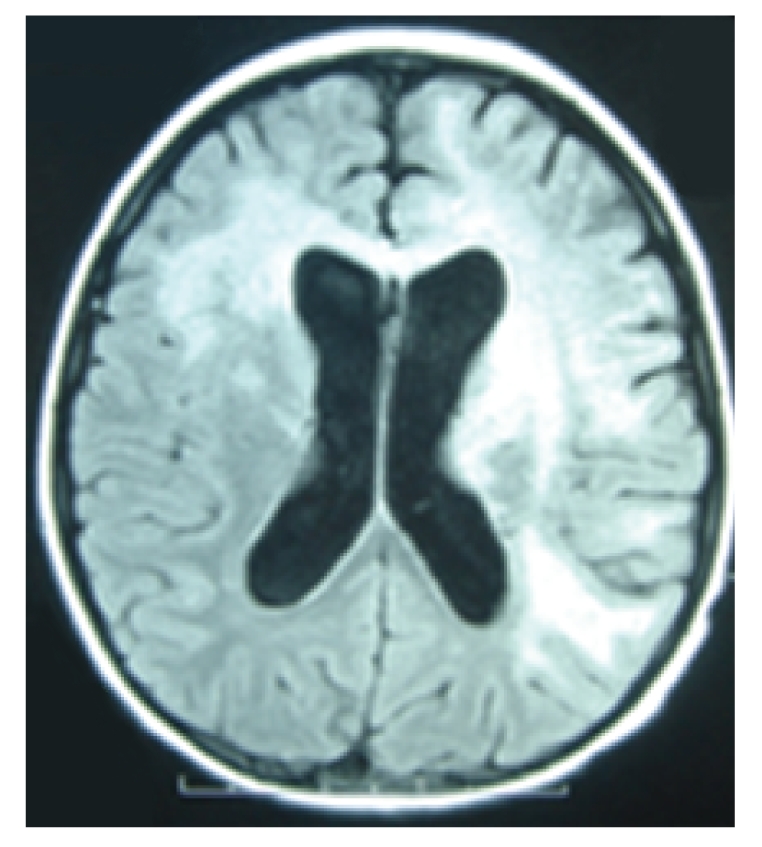
MRI on day 38 of onset of symptoms and 2 weeks post-HAART initiation.

Three days after admission a brain computerized tomography (CT) scan was performed and showed frond-like hypodense lesion at the left frontal lobe with mild effacement of the left frontal horn of the lateral ventricle. Highly active antiretroviral therapy (HAART) regimen was subsequently started 10 days after admission, consisting of GPOvir-Z (coformulated zidovudine 250 mg, lamivudine 150 mg, and nevirapine 200 mg) [3]. Clinically, the patient deteriorated during the 1st and 2nd weeks of HAART with fever and increased right leg weakness, but immunologic and virologic improvement was seen (CD4 T-cell count 0.8%, 10 cells/*μ*L, and a plasma HIV RNA viral level of 26 532 copies/mL, log 4.42). CT (Figure 1) and brain magnetic resonance imaging (MRI; Figure 2) scans were subsequently performed. The scans showed progressive of white matter lesions with asymmetrical deep and subcortical white matter lesions over the left frontotemporoparietal region and the right frontal lobe. The lesion on the left hemisphere involved internal capsule, lentiform nucleus, thalamus, and genu of corpus callosum and anterior cerebellar hemisphere. There were no enhanced areas after the contrast study.

The patient was discharged 7 weeks after the first admission. Upon discharge he was able to walk with assistance, but was unable to speak.

### 2.2. Second Admission

**Figure 3:**
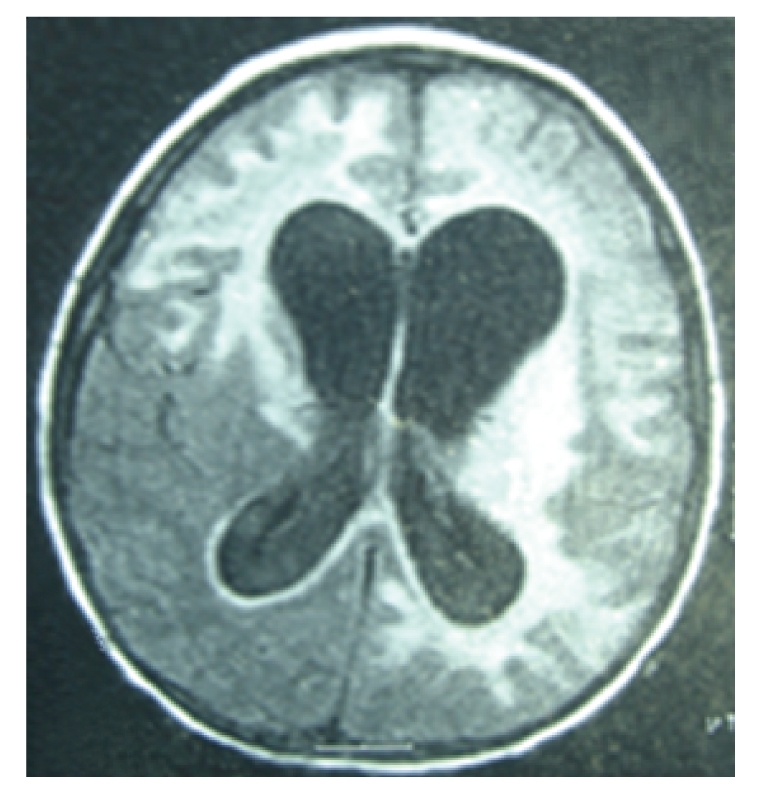
2nd MRI, 3 1/2 months after onset of symptoms, 3 days post-HAART cessation.

The patient was readmitted one day after discharge due to autonomic nervous system dysfunction (nausea, vomiting, and loss of bowel and bladder tone). Deep tendon reflexes were 4+ throughout, and Babinski was positive bilaterally. Continued improvement of immunologic (CD4 T-cell count 2.0%, 30 cells/*μ*L) and virologic (HIV RNA level 3220 copies/mL, log 3.51) measures were seen. Due to progressive neurologic symptoms HAART was ceased. The second MRI (Figure 3) scan was performed and showed a progressive lesion in the same regions as described in the previous MRI, but also found new lesions over the midbrain, pons, and medulla predominantly on the left. The patient was discharged approximately 3 weeks after admission.

### 2.3. Third Admission

**Figure 4:**
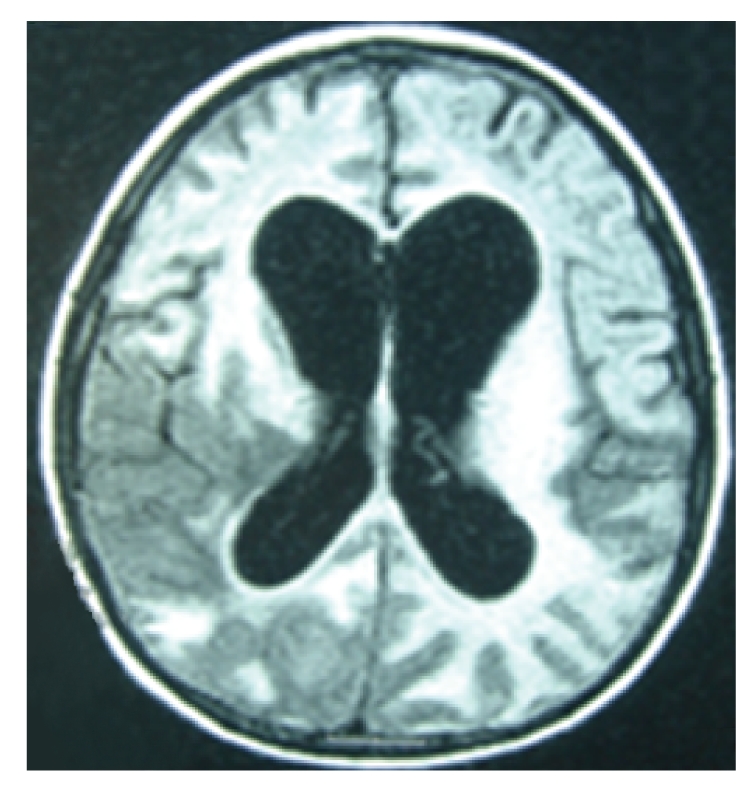
3rd MRI, 4 months after onset of symptoms, 19 days post-HAART cessation.

The patient was readmitted 1 month later (100 days after onset of symptoms). Immune Reconstitution Inflammatory Syndrome (IRIS) was suspected, and the boy was treated with methylprednisolone (2 mg/kg/day) for 5 days. Despite HAART suspension and administration of steroids, his clinical symptoms worsened. The third MRI (Figure 4) scan showed new lesions in the regions of the right brainstem and right hemisphere with gyral enhancement. The patient's mother refused further treatment, he was discharged home, and he subsequently died 1 year later.

## 3. Summary

We report the 2nd case of IRIS associated PML in a perinatally HIV-infected child. Since 1992 there have been reports of 14 HIV-infected children having PML (Table 1) [4–14]. Overall, PML in HIV-infected children has occurred mostly in boys (9/14, 75%), with a median age of 10 years (range: 7–17). Reports have come from Brazil, Hungary, India, Japan, South Africa, Thailand, and USA. Presenting symptoms included: altered speech, hemiplegia, facial palsy, and cerebellar dysfunction. All had significant changes on MRI or CT. Presenting CD4 T-cell counts were low, while viral loads were high. The most common outcome was death.

Neuroimaging is an important part of the diagnosis. Multiple bilateral areas of white matter demyelination without contrast enhancement or mass effect are typical findings. For CT imaging these appear hypodense, while on MRI they have either decreased or increased signals depending on the imaging parameters [14–16]. Treatment for PML is based on HAART initiation or optimization, which has shown improved mortality associated with lower HIV RNA plasma viral levels and higher CD4 T-cell count [17–20].

Although rare, casesof PML associated with IRIS occur where there is clinical deteriorationdespite improvement of immunological and virologicalmeasures after the initiation of HAART [21, 22]. This was seen in the case reported here and the other reported case of PML in an HIV-infected child associated with IRIS [10]. Reported in 2004, a 12-year-old African boy developed cerebellar dysfunction and hemiparesis 5 weeks after starting HAART. He was started on prednisone and continued on HAART. He subsequently had immunologic and virologic improvement with full clinical recovery. Estimates from HIV-infected adults with PML associated IRIS range from 9–19%, typically occurring 3–5 weeks after initiation; this is purportedly much less common in children, as it is only sporadically mentioned in the literature [18, 23]. Therapy for PML associated IRIS has included glucocorticoids in addition to HAART interruption, which have been shown to be both beneficial and to be of no benefit in HIV-infected adults with PML [10, 22, 24–26]. Our patient saw further clinical deterioration, despite a trial of these measures unlike the boy in Africa [10].

PML in HIV-infected adolescents has a wide distribution of ages and geography. Despite the cases presented here, there is limited information about this disease in children. Underdiagnosis is likely to both perpetuate this knowledge gap and discourage physicians from identifying this condition. Additionally, in developing countries, such as Thailand, lack of imaging and laboratory data may further hinder diagnosis. Clinicians should then be cognizant of both of this condition and sequelae after HAART, so that prompt diagnosis and treatment can be made. Clear guidelines would be beneficial to clinicians who face these complex patients.

## Figures and Tables

**Figure 5:**
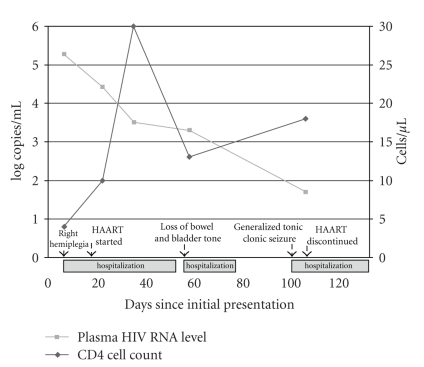
Clinical manifestations and plasma HIV RNA and CD4 cell count levels.

**Table 1:** Overview of studies concerning progressive multifocal leukoencephalopathy in HIV-infected children.

Author	Year reported	Sex	Age (years)	Country	Presentation	MRI/CT	Previous HIV- related event	HAART regimen	CD4 T-cell % or count (cell/*μ*L)		Viral load (copies/mL)		Outcome
									Baseline	Nearest to episode	Baseline	Nearest to episode	
Oberdorfer et al.	2009	M	9	Thailand	Right hemiplegia	Frond-like hypodense lesion at the left frontal lobe	None	Initially nothing then AZT, 3TC, NVP	0.21	0.21	185 976	185 976	Dead
Liptai et al.	2007	M	15 1/2	Hungary	Dizzy, diplopia, clumsy right hand, unsteady gait	Large, nonenhancing lesion of the right cerebellar heimsphere with a slight mass effect	None reported	Initially AZT, ddl then refused HAART	37	10.5	2900	256 000	Dead
Shah and Chudgar.	2005	F	8 1/2	India	Right-sided dystonia, inability to talk, eat, or sit	Asymmetrical subcortical, right frontoparietal, left occipitoparietal and left vasal ganglia lesions	Generalized tonic clonic seizures and loss of consciousness	Initially nothing then AZT, 3TC, EFV, NLF		320			Alive, still has dystonia
Robinson et al.	2004	M	17	USA	Dysarthric speech, facial palsy	Multiple confluent areas of high signal and fluid attenuated inversion recovery in the corona radiata bilaterally	Pneumocystis jiroveci pneumonia	Initially AZT, 3TC then d4T, NVP, lopinavir/ ritonavir then included cidofovir	3	3	10–90 000	29 100	Stable, wide-based gait, dysarthria, right-sided tongue deviation
Nuttall et al.	2004	M	12	South Africa	Acute cerebellar dysfunction, hemiparesis	Nonenhancing low density lesion in the left cerebellar hemisphere	Growth failure, chronic lung disease	d4T, 3TC, efavirenz	1.08	5.45	96 000	Undetectable	Stable, mild cerebellar dysfunction
Inui et al.	1999	M	12	Jaban	Left upper extremity weakness	White matter lesions of the right frontal, parietal and occipital lobes	Candida stomatitis	AZT then ritonavir and 3TC added		9.5		7600	Stable, left hemiparesis
Araujo et al.	1997	M	10	Brazil	Subacute cerebellar dysfunction, dementia	Focal nonenhancing area of low attenuation in the cerebellum	None reported						
Morriss et al.	1997	M	7	USA	Decreased activity, slurred speech, ataxia	Confluent, nonenhancing, low density lesion in the right cerebellar white matter, middle cerebellar peduncle and dorsolateral pons	Candida esophagitis	ddc		0			Death
Whiteman et al.	1993	M	10	USA									
		F	12	USA		Increased signal intensity in the basal ganglia and corona radiata bilaterally							
Berger et al.	1992	F	13	USA	Dysarthria, paresthesias of tongue and chin	Sinusitis and hyperintense signals in the basal ganglia	Oral candidiasis	AZT	421	7			Death
		M	10	USA	Facial palsy	Left frontal lobe lesion	Pneumocystis jiroveci pneumonia	AZT	100				Death
Vandersteenhoven et al.	1992	M	7	USA	Decreased activity, left hemiparesis, falling	Bilateral confluent abnormal white matter hyperintensity in the region of the right subcortical/ periventricular region	None reported	Initially nothing then AZT		390			Death

## Abbreviations

3TC: Lamivudine;
AZT: Ziduvodine;
d4T: Stavudine;
ddC: Zalcitabine;
ddI: Didanosine;
EFV: Efavirenz;
HAART: Highly active antiretroviral therapy;
NLF: Nelfinavir;
NVP: Nevirapine.
